# Effect of Flumethrin on Survival and Olfactory Learning in Honeybees

**DOI:** 10.1371/journal.pone.0066295

**Published:** 2013-06-13

**Authors:** Ken Tan, Shuang Yang, Zhengwei Wang, Randolf Menzel

**Affiliations:** 1 Key Laboratory of Tropical Forest Ecology, Xishuangbanna Tropical Botanical Garden, Chinese Academy of Science, Kunming, China; 2 Eastern Bee Research Institute, Yunnan Agricultural University, Kunming, China; 3 Institut of Biologie and Neurobiology, Freie Universität Berlin, Berlin, Germany; AgroParisTech, France

## Abstract

Flumethrin has been widely used as an acaricide for the control of Varroa mites in commercial honeybee keeping throughout the world for many years. Here we test the mortality of the Asian honeybee *Apis cerana cerana* after treatment with flumethrin. We also ask (1) how bees react to the odor of flumethrin, (2) whether its odor induces an innate avoidance response, (3) whether its taste transmits an aversive reinforcing component in olfactory learning, and (4) whether its odor or taste can be associated with reward in classical conditioning. Our results show that flumethrin has a negative effect on *Apis ceranàs* lifespan, induces an innate avoidance response, acts as a punishing reinforcer in olfactory learning, and interferes with the association of an appetitive conditioned stimulus. Furthermore flumethrin uptake within the colony reduces olfactory learning over an extended period of time.

## Introduction

The ectoparasitic mite species *Varroa destructor* and *Tropilaelaps spp* cause serious damage to apiculture. Acaricides have been widely used for many years in commercial beekeeping to control these mites, and they are known to exert negative effects on honeybees. The acute lethal dose (LD50) of acaricides is age dependent (ranging from 3 to 6 µg per bee) with the lower dose in older bees [1]. Toxic effects have also been observed with lower, sub-lethal acute doses. For example, queens in colonies treated with acaricide-impregnated strips weigh significantly less, have lower ovary weights and show atypical behavior. Furthermore, queen pupae treated with acarcide suffer high mortality rates [2], and even residues in wax can affect the queens’ health adversely [3], [4]. In worker bees, acaricides reduce foraging activity [5].

Pyrethrins and pyrethroids belong to a group of acaricides found in pyrethrum, an extract of certain chrysanthemum flowers [6]. They are often used as insecticides and for controlling insects on pets or livestock. Pyrethrins and pyrethroids affect the insect nervous system by delaying the closing of ion channels, leading to higher neural activity. Increased excitability of neuronal tissue causes fatal nervous system failure and muscle spasms [7], [8].

The pyrethroid *tau*-fluvalinate, a subset of isomers of fluvalinate, was the first synthetic varroacide registered for use in honeybees in the USA [9]. In 1990, plastic strips impregnated with *tau*-fluvalinate (Apistan) replaced homemade plywood strips [10]. According to the label, a single strip contains 0.7 g *tau*-fluvalinate, as much as 10% of which may diffuse from the plastic strip into hive matrices over the course of an 8 week treatment [11], [12]. Like a pyrethroid, *tau*-fluvalinate kills mites by blocking voltage-gated sodium and calcium channels [13]. While most pyrethroids are highly toxic to honeybees, *tau*-fluvalinate is tolerated at high concentrations due in large part to rapid detoxification by cytochrome P450 monooxygenases (P450s) [14]. However, *tau*-fluvalinate also affects the health of reproductive castes in honeybees. Queens exposed to high doses of *tau-*fluvalinate were smaller than untreated queens [2]. Drones exposed to *tau*-fluvalinate during development were less likely to survive to sexual maturity relative to unexposed drones, and they also had reduced weight and lower sperm counts [15].

Flumethrin (C_28_H_22_Cl_2_FNO_3_) is a synthetic pyrethroid ectoparasiticide commonly used in beekeeping. Plastic strips impregnated with 3.6 mg flumethrin are inserted between combs in bee hives so that bees receive topical treatment for varroatosis by contacting the strip [16]. During this exposure, honeybees may directly or indirectly ingest flumethrin through hygienic behaviors during the application period. Furthermore, they may receive low doses of flumethrin through comb wax as they are remodeled after the application period [17].

Flumethrin is also known to exert negative effects on honeybees. The acute lethal doses (LD50) of flumethrin are 0.52 and 0.17 ug per honeybee for 24 and 48 h treatments respectively [1]. Its pharmacological activity is mediated through voltage gated sodium channels as verified by its actions on neural tissue, causing prolonged opening of these channels, leading to a higher sodium influx [18]. Sub-lethal and adverse effects are also caused by its interaction with numerous molecular targets predominantly in the nervous system. The molecular targets include voltage sensitive sodium channels (leading to high frequent repetitive firing followed by a conductive block), molecules of presynaptic terminals (leading to excessive transmitter release), sodium reuptake molecules, GABA receptors, nicotinic acetylcholine receptors, voltage sensitive Ca^2+^ channels, and molecules of the neuroendocrine system [19], [20]. Flumethrin also triggers toxicity effects on reproduction through mechanisms independent of their toxic effects on the nervous system [21]–[25]. These toxic effects may impair important processes involved in behaviour, cognitive functions, and other physiological processes.

Since flumethrin acts on neural functions it is possible that its sub-lethal effects compromise behavior and cognitive abilities. Learning and memory processes enable the bees to respond to the requirements of the colony throughout their life and to deal with highly demanding cognitive actions during foraging (e.g. navigation, learning and remembering cues of the food sources) and during behavior inside the hive (e.g. dance communication, learning in the social context). The impact of acaricides on memory has been suggested already in earlier studies [5]. For instance, Ethyl-parathion, an organophosphate insecticide, appeared to interfere with circadian memory in bees trained to visit a food site at particular times of the day.

So far nothing is known about the sensory reception of flumethrin by honeybees, its potential as a reinforcing stimulus, and its effect on learning and memory retrieval. In this study, we investigated *Apis cerana,* a honeybee species that had no contact with an acaricide since *Varroa destructor* mites are not harmful to them. We ask how this bee species reacts to the odor of flumethrin, whether the odor induces an innate avoidance response, whether its taste transmits a punishing reinforcing component in olfactory learning, and whether flumethrin’s odor (Flu) or taste can be associated with reward in classical conditioning.

## Materials and Methods

Four series of experiments were performed.

### (1) Mortality and Avoidance Responses to Flumethrin

Three *A. c. cerana* colonies were set up in the apiary on the campus of Yunnan Agricultural University, Kunming, China from March to September, 2011. Colonies were comprised of four frames of bees and brood. Thirty worker bees from each colony were trapped at the hive entrance on each test day, immobilized by carbon dioxide, and harnessed in plastic tubes such that they could freely move their antennae and mouthparts [26]. Thirty bees from each colony were harnessed and each bee was fed with 10 µl of 10 µg/g flumethrin-sucrose mixture in 30% sucrose solution, and another 30 bees from each colony were fed with a mixture with a concentration 10 times higher (100 µg/g flumethrin-sucrose mixture). Feeding continued until the bees did not extend their probosces anymore leading to a total of approximately 0.1 ng and 1.0 ng of flumethrin ingested by bees in each treatment respectively. An additional group of 30 bees of each colony provided a control, and these were fed with state the concentration here sucrose solution. These 90 bees were fed three times on each day for 4 days. The number of dead bees was determined every day. Since the bees were equally hungry they consumed approximately the same volume of sucrose solution. Flumethrin used in this study was produced by Best-Reagent.com, Chengdu, China.

We also tested the beeś responses to flumethrin (100 µg/g) diluted in 5% sucrose solution in order to test whether they react aversively to this mixture. A low sucrose concentration was used in order to detect small repellent effects of flumethrin. Thirty hungry bees from each of the three colonies were harnessed in a tube and fed with either this solution or with 5% sucrose solution without flumethrin. The amount of solution taken up from a droplet was measured by a micropipette for each bee.

### (2) Flumethrin as a Reinforcing Component

The paradigm of proboscis-extension response (PER) conditioning [26] was used to study the effect of flumethrin on the rewarding function of sucrose solution. Sixty worker bees from each of the three colonies were trapped at the hive entrance on each test day, immobilized by carbon dioxide, and harnessed in plastic tubes. The bees were kept in an incubator (humidity 65%, temperature 25°C) for 3 hours, and then exposed to odor conditioning. First the beeś PER to sucrose solution (concentration of 30%) was tested, and bees that did not respond (about 50%) were discarded. Bees were then exposed to a puff of *Ageratum* honey odor (Age). Age was made by putting 5 g of *Ageratum* honey on a piece of filter paper which then was transferred into a glass syringe (30 ml). Earlier work has shown that both complex natural odorants and pure volatile components are equally well learned by *Apis mellifera carnica* [26] (see [27] for a recent review). Since we did not know at the outset of our experiments whether *Apis cerana* would show the same behavior we decided in favor of the odorant of nectar this bee species collects from the flowers of *Ageratum*. The bees in our experiments did not taste the honey of *Ageratum* with their antennae or tarsi. Therefore, only the air borne volatiles of *Ageratum* honey, known to be predominantly 5-hydroxymethyl-2-furancarboxaldehyde [28], reached the antennae of the test bee. Bees responding spontaneously to the odor were discarded (<5%), and finally 30 bees from each of the three colonies were used for conditioning.

Odor conditioning was performed as described by Bitterman et al [26]. First the conditioned stimulus (CS, Age) was presented for 5 s and then the unconditioned stimulus (US, 30% sucrose solution) for 3 s. The US was presented by a tooth pick touching the antennae and then the extended proboscis. The US onset was 3 s after CS onset, thus CS and US overlapped by 2 s, and this training trial was repeated five times at an interval of 10 minutes. Three groups were run in parallel. The control group received 5 trials of CS/US and pairing as described. The Flumethrin 100 group received also 5 training trials with the US composed of a mixture of 30% sucrose solution with 100 µg/g flumethrin. In the flumethrin 10 group, a 10 times lower dose of flumethrin (10 ug/g) was present in the sucrose solution.

In another experiment run in parallel with the experiment described below (3) we tested whether flumethrin transmits a punishing component in olfactory conditioning. In order to uncover such an effect it is necessary that the bee responds already to some extent to the conditioned stimulus (Age) before being exposed to flumethrin as a potential punishing stimulus. Therefore, the animals were first exposed to a two trial pre-training (first phase) in which the bees experienced a forward training of Age and sucrose. Then the bees were exposed to a second phase in which they either continued to receive Age paired with sucrose reward for another 4 trials (Age/+), received Age without sucrose reward for another 4 trials (Age/−) testing for extinction phenomena, or they received Age followed by Flu for another 4 trials (Age+Flu/−). The duration of the time interval between Age and Flu is 1 s, and the duration of the flumethrin stimulation is 5 s. Flu was prepared by soaking 5 µl of 100 µg/g flumethrin solution in water on filter paper and then transferring the paper into a glass syringe (30 ml).

### (3) Flumethrin as a CS Component

Fifty bees from each test colony were prepared in a similar way as described above. Depending on the particular experimental group each bee was exposed to the conditioned stimulus for 5 s (Flu alone, Age alone or in sequence) and then to the sucrose reward. Either odor was presented for 5s, first the Flu and then the Age or the other way round. The interval between the two odors was 1 s. US presentation followed the procedure described above.

Each test bee received five conditioning trials at trial intervals of 10 min. The 150 test bees of each colony were divided into 5 groups which were run in parallel. Age/+: CS: Age, US: sucrose; Flu+Age/+: CS: first Flu then Age, US: sucrose; Age/−: CS: Age, no US; Age+Flu/+: CS: Age first then Flu, US: sucrose; Flu/+: CS: Flu odor, US: sucrose.

### (4) Olfactory Conditioning of Animals Exposed to Flumethrin within the Colony

In order to test the long-term effect of flumethrin exposure we treated whole colonies with this acaricide. A flumethrin-sucrose mixture (20 ml 10 µg/g) was sprayed on the combs of each of the three hives every three days for two weeks following the procedure of flumethrin application as described in the Introduction. Then 30 bees from each colony were prepared for PER conditioning as described above, using Age as the conditioned stimulus and sucrose solution as the reward (Flu treatment). As a first control we tested bees from each colony for their performance in PER conditioning before the respective colony was treated with the flumethrin-sucrose mixture (Flu pre-treatment). In the second test group we ran the same PER conditioning experiments 2 weeks after termination of the flumethrin-sucrose treatment (Flu post-treatment).

Statistics: Chi-square tests were applied to analyse data of the mortality tests. The independent t-test was used in the aversion tests. Repeated measure ANOVA or multivariate ANOVA were applied to examine the acquisition functions of bees [29]. We used LSD tests to determine if there were any differences among different groups. All tests were performed using Statistica version 10 (Statsoft Inc, 2011).

## Results

### (1) Mortality and Avoidance Responses to Flumethrin

The results of the mortality tests were: 62.1±2.63% mortality in the flumethrin 100 group (24 hours after the bees were fed with 100 µg/g flumethrin*/*sucrose solution), and 59.21±9.21% in the flumethrin 10 group (with 10 ug/g flumethrin*/*sucrose solution). These values are significantly higher than those from the control group fed with sucrose solution without flumethrin(22.20±3.6%)(χ_1_^2^ = 32.84, df = 1, P<.001;χ_2_^2^ = 28.41, df = 1, P<.001). No difference was found between the two test groups (χ^2^ = 0.19, df = 1, P = 0.66). All bees of the two test groups died within 72 hours, but only 57±5% died in the control group. The difference is highly significant (χ^2^ = 54.78, df = 1, P<.001) (Table 1). Thus bees ingesting flumethrin had a higher mortality rate than bees fed sucrose solution without flumethrin.

**Table 1:** Means±SE of bee mortality when fed with 10 ug/g f*lumethrin* sucrose (Flumethrin 10 group) or 100 ug/g (Flumethrin 100 group) *Flumethrin* sucrose solution and control group (each group n = 30).

	Mortality %			
	Day 1	Day 2	Day 3	Day 4
Control	21.79^a^±5.88	45.07^a^±0.39	56.99^a^±4.72	71.11^a^±9.75
Flumethrin10 group	59.21^b^±9.21	88.42^b^±1.58	100^b^±0	100^b^±0
Flumethrin100 group	62.09^b^±2.63	88.83^b^±2.34	100^b^±0	100^b^±0

Avoidance responses to flumethrin: Each bee of the test group (n = 90) ingested on average 8.26±3.29 µl of sucrose solution mixed with flumethrin (100 µg/g), while each bee of the control group (n = 90) ingested 21.31±5.19 µl of pure sucrose solution, a highly significant difference (t = 20.16, P<.001), indicating that bees avoid flumethrin, and therefore must taste it in sucrose solution.

### (2) Flumethrin as a Reinforcing Component

**Figure 1:**
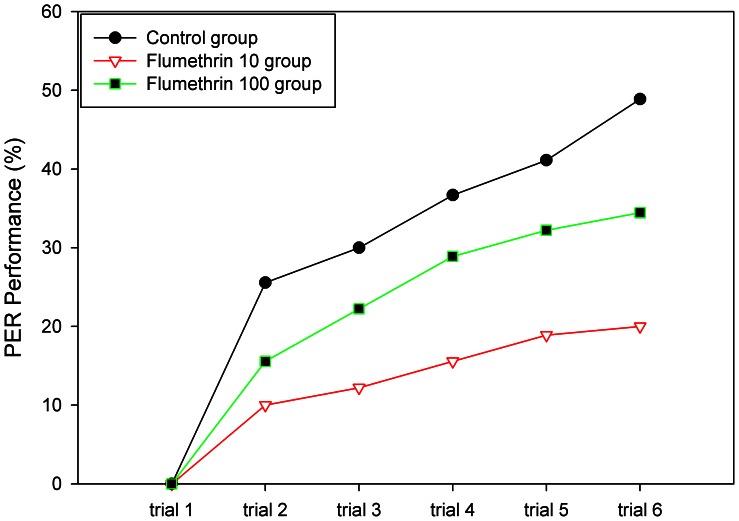
The effect of flumethrin as a reinforcing component. Three groups were tested with *Age* as the CS in all three groups. The control group received 30% sucrose solution as US, the flumethrin 10 group received 10 µg/g of flumethrin-sucrose solution as US, the flumethrin 100 group received 100 µg/g of flumethrin-sucrose solution as US. Number of animals in each group (n = 90).

Here we first asked whether flumethrin reduces the reward property of sucrose solution in olfactory conditioning. Age was the CS in all three groups. The control group was conditioned in the standardized way (see Methods) with 30% sucrose solution as US, the flumethrin 10 group received 10 µg/g of flumethrin-sucrose solution as US, and flumethrin 100 group received 100 µg/g of flumethrin-sucrose solution as US (Fig. 1). Acquisition was highest in the control group and lowest in the flumethrin 10 group. Significant differences were found between the three respective acquisition groups (repeated measure ANOVA, group effect: F_2,6_ = 48.14, P = .002), Post hoc tests showed that both flu 10 and 100 groups differed from the control, and the flumethrin 10 group also showed significantly lower performance than the flumethrin 100 group(LSD: P<0.05). In all three groups, and even when the reward contained flumethrin, acquisition was significantly lower, as shown by the significant trial effect (ANOVA, F_5,30_ = 109.23, P<.001). However, a significant group× trial interaction (F_10, 30_ = 5.97, P<.001) indicated that flumethrin significantly affected the course of acquisition.

**Figure 2:**
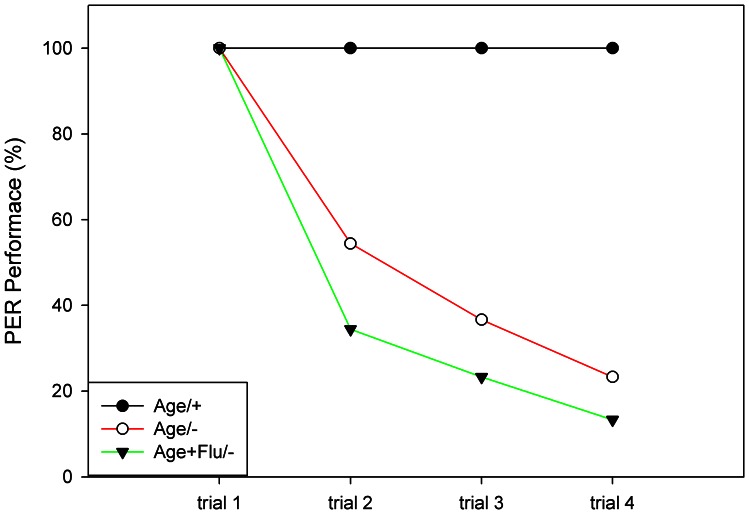
Flumethrin transmits an inhibitory (punishing) component in PER conditioning. The figure shows the conditioned responses in the second phase of the experiment. Age/+ continued to receive sucrose as a reward, Age/− received no sucrose reward anymore (extinction), and Age+Flu/− received Age then followed by Flu, without sucrose reward. Number of animals in each group (n = 90).

In a parallel experiment we tested whether flumethrin transmits an aversive component in PER conditioning. In the first phase, all bees were conditioned to *Age* by forward pairing twice with sucrose solution. Then the bees responding to Age alone during the second trial were split into three groups which differed with respect to the reinforcing stimulus during the second phase (Age/+: Age pair with sucrose solution; Age/−: no sucrose solution, Age alone; Age+Flu/−: Age followed by Flu, without reward). We found significant differences in the second phase of the experiment between the three groups (Fig. 2, repeated measure ANOVA, group effect: F_2, 6_ = 746.57,P<0.01). The conditioned responses decrease significantly in group Age/− (the Age extinction test group) and Age+Flu/− (repeated measure ANOVA, trial effect: F_3, 18_ = 1158.42, P<.01). A significant group× trial interaction confirmed that the evolution of responses was different in the three groups (F_6, 18_ = 299.17, P<0.01). Responses in both Age/− and Age+flu/− groups were lower than in the Age/+ group (LSD: P<0.01). In addition, performances were even lower in the Age+flu/− group compared to the Age/− group (LSD: P<0.01), showing that flumethrin transmits a punishing reinforcing component.

### (3) Flumethrin as a CS Component

**Figure 3:**
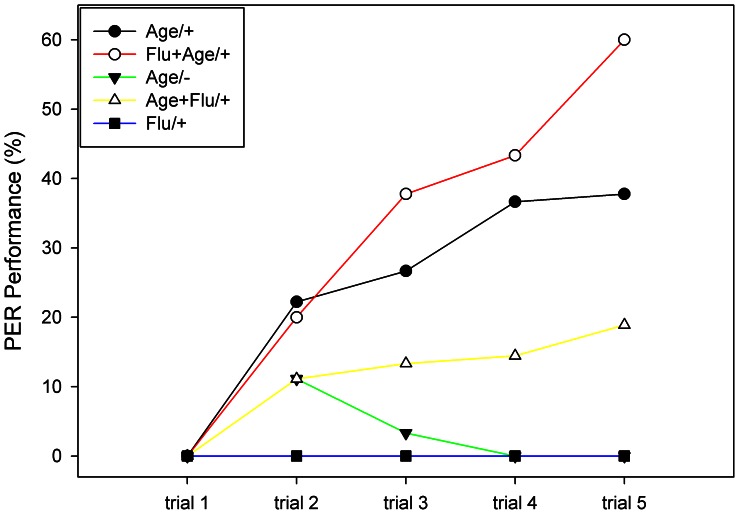
Flumethrin as a component of the CS or US. Five groups were run in parallel. Age +: CS: Age, US: sucrose; Flu+Age/+: CS: first Flu then Age, US: sucrose; Age/−: CS: Age, no US; Age+Flu/+: CS: Age first then Flu, US: sucrose; Flu/+: CS: Flu, US: sucrose. Number of animals in each group (n = 90).

Since it is unknown whether the reception of flumethrin requires contact chemoreceptors or can also be received as air born stimulus we next asked whether flumethrin as a component of the CS effects olfactory learning (note that Flu was provided here as an airborne stimulus, see Methods). If this is the case one needs to ask whether flumethrin as a CS component provides an inhibitory component reducing the association of Age with reward. The CS was either a single odor (Age or Flu) or a sequence of the two odors. The sequential appearance of the two odors allows also for testing whether flumethrin has a hidden punishing component as an US because sequential CSs allow to test whether their associability differs. In such a test it is necessary to control for the sequence effect. Five groups were run in parallel. Age/+ (control group): the CS was only Age and the US a 30% sucrose solution; Flu+Age/+: the CS consisted first of Flu followed by Age, the US was 30% sucrose solution; Age/−: the CS was Age, and no US was given; Age+Flu/+: the CS consisted of Age first followed by Flu, and the US was sucrose solution; Flu/+: the CS was Flu, and the US: sucrose solution (Fig. 3). Age/+ provides the reference for the different conditioning procedures, Flu+Age/+ and Age+Flu/+ tested for a potential deterrent CS effect of flumethrin either in a forward or backward sequence with Age, Age/− serves as control for the associative effect, and Flu/+ tested for flumethrin as a CS. Surprisingly the highest acquisition was found for Flu+Age/+ followed by the acquisition of Age/+. No acquisition was seen for Age/− (the Age extinction test group) which is not surprising since Age was not paired with sucrose reward. The finding that the Flu/+ treatment also showed no learning indicates that flumethrin may either not be detected as a CS because it is not sensed or its deterrent effect compensates the appetitive effect of sucrose reward. The two groups with intermediate acquisition are Age/+ and Age+Flu/+, so the two rewarded groups in which Age started the odor sequence. Repeated measure ANOVA showed a significant difference among these five groups (group effect: F_4, 10_ = 108.16, P<0.001). When analyzing the trial effect we found significant differences (F_4, 40_ = 389.20, P<0.001) as well as in a trial × group interaction effect (F_16, 40_ = 112.15, P<.001). Statistical significantly differences were observed among these five groups via post hoc tests (LSD: P<0.01). While the Flu/+ group did not lead to acquisition and Age/− group lead to low performance, we compared the other three groups. The Flu+Age/+ group reached about 60% which was higher than in the Age+Flu/+ group with only 20% in the fifth trial, and even higher than in the Age/+ group (LSD: P<0.01). The significantly higher response to a sequence of first Flu and then Age as a CS is surprising, and may indicate an announcement of release of an aversive stimulus (flumethrin) by the following Age treatment. This possibility requires further studies.

### (4) Olfactory Conditioning of Animals Exposed to Flumethrin within the Colony

**Figure 4:**
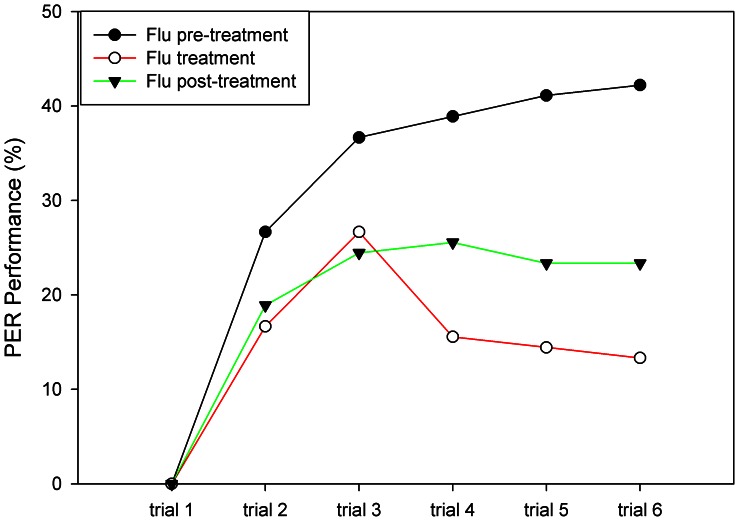
Olfactory conditioning of animals exposed to flumethrin within the colony. Flu pre-treatment (control): PER conditioning before the flumethrin-treatment period; Flu treatment: PER conditioning during the flumethrin-treatment period; Flu post-treatment: PER conditioning two weeks after the flumethrin-treatment period. Number of animals in each group (n = 90).

Finally we asked whether animals exposed to flumethrin within the colony by spraying flumethrin-sucrose mixtures on the combs differ with respect to olfactory PER conditioning (Fig. 4). We found significant differences of PER conditioning among the three groups tested (F_2, 6_ = 21.38, p = .002) (Flu pre-treatment: no flumethrin treatment; Flu treatment: flumethrin was sprayed into the colony; Flu post-treatment: two weeks after flumethrin was sprayed into the colony). Bees of the Flu treatment and Flu post-treatment groups showed significantly lower PER performance than the control group (Flu pre-treatment) (trial effect: F_5, 30_ = 194.17, P<.001, trial group interaction: significant, F_10, 30_ = 19.32, P<.001). Thus take-up of flumethrin within the colony over an extended period of time reduces learning performance as tested in PER conditioning, and the effect is not lost after 2 weeks.

## Discussion

Our results clearly show that flumethrin compromises life span and induces avoidance responses. It also has an inhibitory effects on reward learning both as a component of the learned stimulus and as a reinforcing stimulus.

Flumethrin is directed against the pest mite *Varroa*, but it also affects the health of honeybees. Since flumethrin acts on voltage gated sodium channels [18] and interacts with several molecular targets [19], [20] it may impair physiological processes and cognitive functions. Sensory perception, neural integration and synaptic plasticity underlying learning and memory formation depend on voltage-gated and transmitter- as well as second messenger- controlled ion channels in the honeybee brain [30]. It is thus likely that any modulation or blocking of ion channels or receptors will interfere with these neural processes. Since pyrethroids lead to high frequency spiking and interfere with nicotinic acetylcholine receptors [19], [20] it is likely that they have detrimental effects on high order processing and synaptic plasticity. Avoidance responses of honeybees were found for synthetic pyrethroids [31], [32], but it was unknown whether contact chemoreception is required and whether the deterrent effect interferes with appetitive learning. Since all of the studies on the effect of synthetic pyrethroids on honeybees were performed with *Apis mellifera* we needed to test first how extended treatment with flumethrin within the colony reduces survival rate. Our results confirm that *Apis cerana* avoids flumethrin and suffers from reduced survival rates. Most importantly we found that extended treatment with flumethrin inside the hive interferes with learning performance, suggesting a previously unknown effect on brain metabolism essential for learning.

The goal of our study was to characterize the behavioral components of this detrimental effect rather than to unravel the cellular processes involved in learning related neural processes. In our context it was, therefore, necessary to establish first whether flumethrin can be perceived not only by contact chemoreception but also as air borne stimulus. In such a case one needs to consider not only the avoidance inducing potential but also the possibility of a punishing component as a unconditioned stimulus (US) received by contact chemoreception in a learning situation but also the role as a conditioned olfactory stimulus (CS). We have applied the PER conditioning paradigm in which the animal is exposed to a olfactory CS followed by an appetitive (rewarding) US. This paradigm does not only mimic olfactory learning of foragers during their flower visiting activities but also olfactory reward learning inside the hive [33].

The punishing property of flumethrin is reflected by several aspects, as shown by our PER conditioning experiments. Flumethrin transmits a punishing reinforcing component when mixed with the appetitive sucrose reward, and it leads to stronger extinction as compared to extinction tests without sucrose reward and without flumethrin. The deterrent and possibly also a punishing component of flumethrin can also be observed in experiments in which it is a component of the CS. When two CSs are applied in sequence (Age and Flu) the resulting acquisition function depends on the temporal order of these two CSs (Fig. 3). These effects can be understood as resulting from a punishing component of flumethrin interacting with an appetitive component of Age. When bees are conditioned to a sequence of two odors the odor closest to the rewarding US will dominate the conditioned response because it is acquired more effectively [34]. Since conditioned responses were lower when flumethrin was closer to the US, and were higher when Age was closer to US the two CSs differ in their associability; Age had a higher associability than flumethrin. Interestingly acquisition was higher for the CS sequence flumethrin- Age than in the control group (only Age). This effect can be understood on the assumption that flumethrin signals a punishing component because the following association of Age with reward signals a relief from punishment, and the animals respond more strongly to the last CS, Age. Such relief from punishment has been found in *Drosophila* in a backward conditioning paradigm which simulates the induction of a reversal of CS value as compared to forward pairing [35]. Similar responses have been found in mammals and humans [36]. This interpretation is supported by our finding that forward pairing of Flu with sucrose reward does not lead to an acquisition of Flu (Fig. 3, Flu/+). Since Flu is recognized, as shown by the effects in the other groups of Fig. 3, there are two mutually counteracting components work together compensating each other: the punishing effect of flumethrin and the forward pairing effect of flumethrin/sucrose reward.

Several insecticides were tested for their effects on learning in honeybees. Different pyrethroids were found to interfere with associative learning both under field and laboratory conditions [37], [38]. The phenylpyrazole fipronil causes reduced learning and memory retention as measured with the olfactory PER conditioning paradigm [38], [39]. Bees were exposed to sub-lethal doses of fipronil for 11 days and the surviving bees were tested. Different treatments with fipronil elicited different effects. Injection of a low dose impaired olfactory memory, and a higher dose applied to the thorax had no observable effect but altered the side-specificity of antennal tactile learning [40], [41]. In our study, prolonged exposure to flumethrin within the colony led to a marked decrease in olfactory PER conditioning, and this effect was not compensated for in an interval of two weeks without flumethrin exposure. It is therefore likely that chronic exposure to flumethrin induces irreproducible damage to the neural circuitry underlying olfactory learning.

Taken together our data show that flumethrin is not only avoided but also reduces life span within short and extended periods. Most importantly it also interferes with learning related processes. These processes can be traced to two functions, a deterrent component as a conditioned stimulus and an aversive reinforcing effect leading to a form of punishment of the conditioned stimulus received shortly before. It is, therefore, likely that flumethrin has a substantial impact on the social life of honeybees since many aspects of social interactions are controlled by learning processes.

## References

[1] Johnson RM, Ellis MD, Mullin CA, Frazier M (2010) Pesticides and honey bee toxicity–USA. Apidologie 41: 312–331.

[2] Haarmann T, Spivak M, Weaver D, Weaver B, Glenn T (2002) Effects of fluvalinate and coumaphos on queen honey bees (Hymenoptera: Apidae) in two commercial queen rearing operations. Journal of Economic Entomology 95: 28–35. 11942761 10.1603/0022-0493-95.1.28

[3] Collins AM, Pettis JS, Wilbanks R, Feldlaufer MF (2004) Performance of honey bee (*Apis mellifera*) queens reared in beeswax cells impregnated with coumaphos. Journal of Apicultural Research 43: 128–134.

[4] Pettis JS, Collins AM, Wilbanks R, Feldlaufer MF (2004) Effects of coumaphos on queen rearing in the honey bee, *Apis mellifera* . Apidologie 35: 605–610.

[5] Schneider CW, Tautz J, Grünewald B, Fuchs S (2012) RFID tracking of sublethal effects of two neonicotinoid insecticides on the foraging behavior of *Apis mellifera* . PLoS One 7: e30023. 22253863 10.1371/journal.pone.0030023 PMC3256199

[6] Leahey JP (1985) The pyrethroid insecticides. Philadelphia, PA: Taylor & Francis.

[7] Klaassen CD, Watkins JB (1999) Casarett and Doull's toxicology: the basic science of poisons. Toronto, Canada: McGraw-Hill.

[8] Costa L (1997) Human health effects of pestcides, Ocupational medicine, State of the art reviews. In: MG K, editor. Basic Toxicology of Pesticides. Philadelphia, PA: Hanley and Belfus. 251–268. 9220485

[9] Ellis M, Nelson R, Simonds C (1988) A comparison of the fluvalinate and ether roll methods of sampling for Varroa mites in honey bee colonies. American Bee Journal 128: 262–263.

[10] PAN (2009) Pesticide action network. http://www.pesticideinfo.org.

[11] Bogdanov S, Kilchenmann V, Imdorf A (1998) Acaricide residues in some bee products. Journal of Apicultural Research 37: 57–67.

[12] VitaEuropeLtd. (2009) ApiGuard. http://www.vita-europe.com.

[13] Davies T, Field L, Usherwood P, Williamson M (2008) DDT, pyrethrins, pyrethroids and insect sodium channels. IUBMB life 59: 151–162. 10.1080/15216540701352042 17487686

[14] Johnson RM, Wen Z, Schuler MA, Berenbaum MR (2006) Mediation of pyrethroid insecticide toxicity to honey bees (Hymenoptera: Apidae) by cytochrome P450 monooxygenases. Journal of Economic Entomology 99: 1046–1050. 16937654 10.1603/0022-0493-99.4.1046

[15] Rinderer TE, Guzman LI, Lancaster VA, Delatte GT, Stelzer JA (1999) Varroa in the mating yard. I. The effects of *Varroa jacobsoni* and apistan on drone honey bees. American Bee Journal 139: 134–139.

[16] EMEA (1998) Summary report, committee for veterinary medicinal products, flumethrin. London, United Kingdom: The European Agency for the Evaluation of Medicinal Products.

[17] Oruc H, Hranitz J, Sorucu A, Duell M, Cakmak I, et al (2012) Determination of Acute Oral Toxicity of Flumethrin in Honey Bees. Journal of Economic Entomology 105: 1890–1894. 23356050 10.1603/ec12055

[18] Zlotkin E (1999) The insect voltage-gated sodium channel as target of insecticides. Annual Review of Entomology 44: 429–455. 10.1146/annurev.ento.44.1.429 9990721

[19] Sattelle DB, Yamamoto D (1988) Molecular targets of pyrethroid insecticides. Adv Insect Physiol 20: 147–213.

[20] Soderlund DM, Bloomquist JR (1989) Neurotoxic actions of pyrethroid insecticides. Annual Review of Entomology 34: 77–96. 10.1146/annurev.en.34.010189.000453 2539040

[21] Yousef MI (2010) Vitamin E modulates reproductive toxicity of pyrethroid lambda-cyhalothrin in male rabbits. Food and Chemical Toxicology 48: 1152–1159. 20138202 10.1016/j.fct.2010.02.002

[22] Zhang H, Wang H, Wang Q, Zhao XF, Liu P, et al (2010) Pubertal and early adult exposure to fenvalerate disrupts steroidogenesis and spermatogenesis in mice at adulthood. Journal of Applied Toxicology 30: 369–377. 20063364 10.1002/jat.1507

[23] Zhang H, Wang H, Ji YL, Zhang Y, Yu T, et al (2010) Maternal fenvalerate exposure during pregnancy persistently impairs testicular development and spermatogenesis in male offspring. Food and Chemical Toxicology 48: 1160–1169. 20138952 10.1016/j.fct.2010.02.003

[24] Joshi SC, Bansal B, Jasuja ND (2011) Evaluation of reproductive and developmental toxicity of cypermethrin in male albino rats. Toxicological & Environ Chemistry 93: 593–602.

[25] Zhao XF, Wang Q, Ji YL, Wang H, Liu P, et al (2011) Fenvalerate induces germ cell apoptosis in mouse testes through the Fas/FasL signaling pathway. Archives of toxicology 85: 1101–1108. 21279716 10.1007/s00204-011-0654-9

[26] Bitterman M, Menzel R, Fietz A, Schaefer S (1983) Classical conditioning of proboscis extension in honeybees (*Apis mellifera*). J Comp Psychol 97: 107–119. 6872507

[27] Matsumoto Y, Menzel R, Sandoz JC, Giurfa M (2012) Revisiting olfactory classical conditioning of the proboscis extension response in honey bees: a step towards standardized procedures. Journal of Neuroscience Methods 211: 159–167. 22960052 10.1016/j.jneumeth.2012.08.018

[28] Chen Y, He S, Dong X, Yang Y, Pu Y, et al (2007) Studies on the Components of Volatile from the Honey of *Elsholtzia ciliate*(Thuab) Hyland. Journal of Yunnan Agricultural University 22: 309–312.

[29] Mota T, Giurfa M, Sandoz JC (2011) Color modulates olfactory learning in honeybees by an occasion-setting mechanism. Learning & Memory 18: 144–155. 21330377 10.1101/lm.2073511

[30] Grünewald B, Anna Wersing A, Wüstenberg D (2004) Learning channels. Cellular physiology of odor processing neurons within the honeybee brain. Acta Biologica Hungarica 55: 53–63. 15270218 10.1556/ABiol.55.2004.1-4.7

[31] Taylor KS, Waller GD, Crowder LA (1987) Impairment of a classical conditioned response of the honey bee (*Apis mellifera* L.) by sublethal doses of synthetic pyrethroid insecticides. Apidologie 18: 243–252.

[32] Mamood AN, Waller GD (2008) Recovery of learning response by honeybees following a sublethal exposure to permethrin. Physiological Entomology 15: 55–60.

[33] Farina WM, Grüter C, Acosta L, Mc Cabe S (2007) Honeybees learn floral odors while receiving nectar from foragers within the hive. Naturwissenschaften 94: 55–60. 17021915 10.1007/s00114-006-0157-3

[34] Menzel R, Giurfa M (2006) Dimensions of cognition in an insect, the honeybee. Behavioral and Cognitive Neuroscience Reviews 5: 24–40. 16816091 10.1177/1534582306289522

[35] Yarali A, Krischke M, Michels B, Saumweber T, Mueller MJ, et al (2009) Genetic distortion of the balance between punishment and relief learning in Drosophila. Journal of Neurogenetics 23: 235–247. 19052955 10.1080/01677060802441372

[36] Andreatta M, Mühlberger A, Yarali A, Gerber B, Pauli P (2010) A rift between implicit and explicit conditioned valence in human pain relief learning. Proceedings of the Royal Society B: Biological Sciences 277: 2411–2416. 20356893 10.1098/rspb.2010.0103 PMC2894900

[37] Decourtye A, Devillers J, Cluzeau S, Charreton M, Pham-Delègue MH (2004) Effects of imidacloprid and deltamethrin on associative learning in honeybees under semi-field and laboratory conditions. Ecotoxicology and Environmental Safety 57: 410–419. 15041263 10.1016/j.ecoenv.2003.08.001

[38] Decourtye A, Devillers J, Genecque E, Menach KL, Budzinski H, et al (2005) Comparative sublethal toxicity of nine pesticides on olfactory learning performances of the honeybee *Apis mellifera* . Archives of Environmental Contamination and Toxicology 48: 242–250. 15750780 10.1007/s00244-003-0262-7

[39] Aliouane Y, el Hassani AK, Gary V, Armengaud C, Lambin M, et al (2009) Subchronic exposure of honeybees to sublethal doses of pesticides: effects on behavior. Environmental Toxicology and Chemistry 28: 113–122. 18700810 10.1897/08-110.1

[40] El Hassani AK, Dupuis JP, Gauthier M, Armengaud C (2009) Glutamatergic and GABAergic effects of fipronil on olfactory learning and memory in the honeybee. Invertebrate Neuroscience 9: 91–100. 19851797 10.1007/s10158-009-0092-z

[41] Bernadou A, Démares F, Couret-Fauvel T, Sandoz J, Gauthier M (2009) Effect of fipronil on side-specific antennal tactile learning in the honeybee. Journal of Insect Physiology 55: 1099–1106. 19723527 10.1016/j.jinsphys.2009.08.019

